# Classical swine fever virus failed to activate nuclear factor-kappa b signaling pathway both *in vitro* and *in vivo*

**DOI:** 10.1186/1743-422X-9-293

**Published:** 2012-11-27

**Authors:** Li-Jun Chen, Xiao-Ying Dong, Ming-Qiu Zhao, Hai-Yan Shen, Jia-Ying Wang, Jing-Jing Pei, Wen-Jun Liu, Yong-Wen Luo, Chun-Mei Ju, Jin-Ding Chen

**Affiliations:** 1 College of Veterinary Medicine, South China Agricultural University, 483 Wu Shan Road, Guangzhou, Tian He District, 510642, China

**Keywords:** CSFV, NF-κB, PK-15, PBMC, p65, IκBα

## Abstract

**Background:**

Classical swine fever virus (CSFV) is the cause of CSF which is a severe disease of pigs, leading to heavy economic losses in many regions of the world. Nuclear factor-kappa B (NF-κB) is a critical regulator of innate and adaptive immunity, and commonly activated upon viral infection. In our previous study, we found that CSFV could suppress the maturation and modulate the functions of monocyte-derived dendritic cells (Mo-DCs) without activating NF-κB pathway. To further prove the effects of CSFV on the NF-κB signaling pathway, we investigated the activity of NF-κB after CSFV infection *in vivo* and *in vitro*.

**Methods:**

Attenuated Thiverval strain and virulent wild-type GXW-07 strain were used as challenge viruses in this study. Porcine kidney 15 (PK-15) cells were cultured *in vitro* and peripheral blood mononuclear cells (PBMCs) were isolated from the blood of CSFV-infected pigs. DNA binding of NF-κB was measured by electrophoretic mobility shift assays (EMSA), NF-κB p65 translocation was detected using immunofluorescent staining, and p65/RelA and IκBα expression was measured by Western Blotting.

**Results:**

Infection of cells with CSFV *in vitro* and *in vivo* showed that compared with tumor necrosis factor alpha (TNF-α) stimulated cells, there was no distinct DNA binding band of NF-κB, and no significant translocation of p65/RelA from the cytoplasm to the nucleus was observed, which might have been due to the apparent lack of IkBa degradation.

**Conclusions:**

CSFV infection had no effect on the NF-κB signaling pathway, indicating that CSFV could evade host activation of NF-κB during infection.

## Background

CSFV, together with Bovine viral diarrhoea virus (BVDV) and sheep Border disease virus (BDV), belongs to the genus *Pestivirus*, the family *Flaviviridae*[1]. CSFV is known to have high affinity to cells of the immune system, which seems to relate to its detrimental effects on the immune and hematopoietic systems [2]. Furthermore, CSFV can efficiently evade and compromise the host immune system, causing a severe disease of pigs characterized by fever, hemorrhage, thrombocytopenia, lymphoid organ atrophy and severe lymphopenia, particularly in B cells, due to the apoptosis of uninfected lymphocytes [3,4]. The lymphopenia can result in immunosuppression and serves as a hallmark of CSFV infections. *Pestiviruses* such as CSFV and BVDV can also cross the placenta to infect the developing fetus, resulting in the birth of persistently infected animals [5,6]. Recently, studies have found that CSFV evolves mechanisms for the inhibition of inflammation and type I interferon (IFN) production in infected cells, preventing double-stranded RNA-mediated apoptosis and giving rise to long-term infections [7,8]. Although a large number of works have been done, the mechanism of the CSFV infection *in vivo* and *in vitro* has not been fully elucidated.

Viruses have evolved sophisticated control mechanisms to redirect the cellular signal transduction pathway to its own advantage, and NF-κB pathway is a common target of many viruses [9]. The NF-κB pathway is recognized to be the central mediator of immune responses in mammals [10], because several proteins encoded by target genes of NF-κB participate in the activation of immune and inflammatory responses. Therefore, NF-κB activation during viral infection has been interpreted as a protective response of the host to the viral pathogen [11]. Interestingly, certain viruses can utilize the activation of NF-κB as a strategy to increase viral replication and viral progeny production [12]. Whereas other reports indicate that some viruses inhibit the NF-κB pathway [13-18]. The suppression of NF-κB activation represents a potential strategy of viral escape from the host immune system and contributes to virus pathogenesis in infected cells.

NF-κB is also a host nuclear transcription factor, activated by multiple stimuli including inflammatory cytokines, growth factors, bacterial and viral infections, and plays an important role in inflammation, innate immune responses, regulation of cell proliferation and survival [11,19,20]. In non-stimulated cells, NF-κB is sequestered as a cytoplasmic complex, whose predominant form is a heterodimer consisting of p50 and p65 (RelA) subunits, bound to inhibitory IκB proteins [21,22]. Stimulation of cells by diverse agents causes phosphorylation of IκB and its degradation by the proteosome subsequently [23]. Liberated NF-κB is transported into the nucleus where it induces the transcription of target genes, including IκBα acting as the major feedback inhibitor of the NF-κB pathway [24].

In the present study, we investigated NF-κB activity *in vivo* and *in vitro* in order to elucidate how CSFV carried out its infection in the host. The results demonstrate that CSFV does not activate the NF-κB signaling pathway which may represent a mechanism of CSFV-induced immunomodulation.

## Results

### DNA binding activity of NF-κB by EMSA analysis

**Figure 1:**
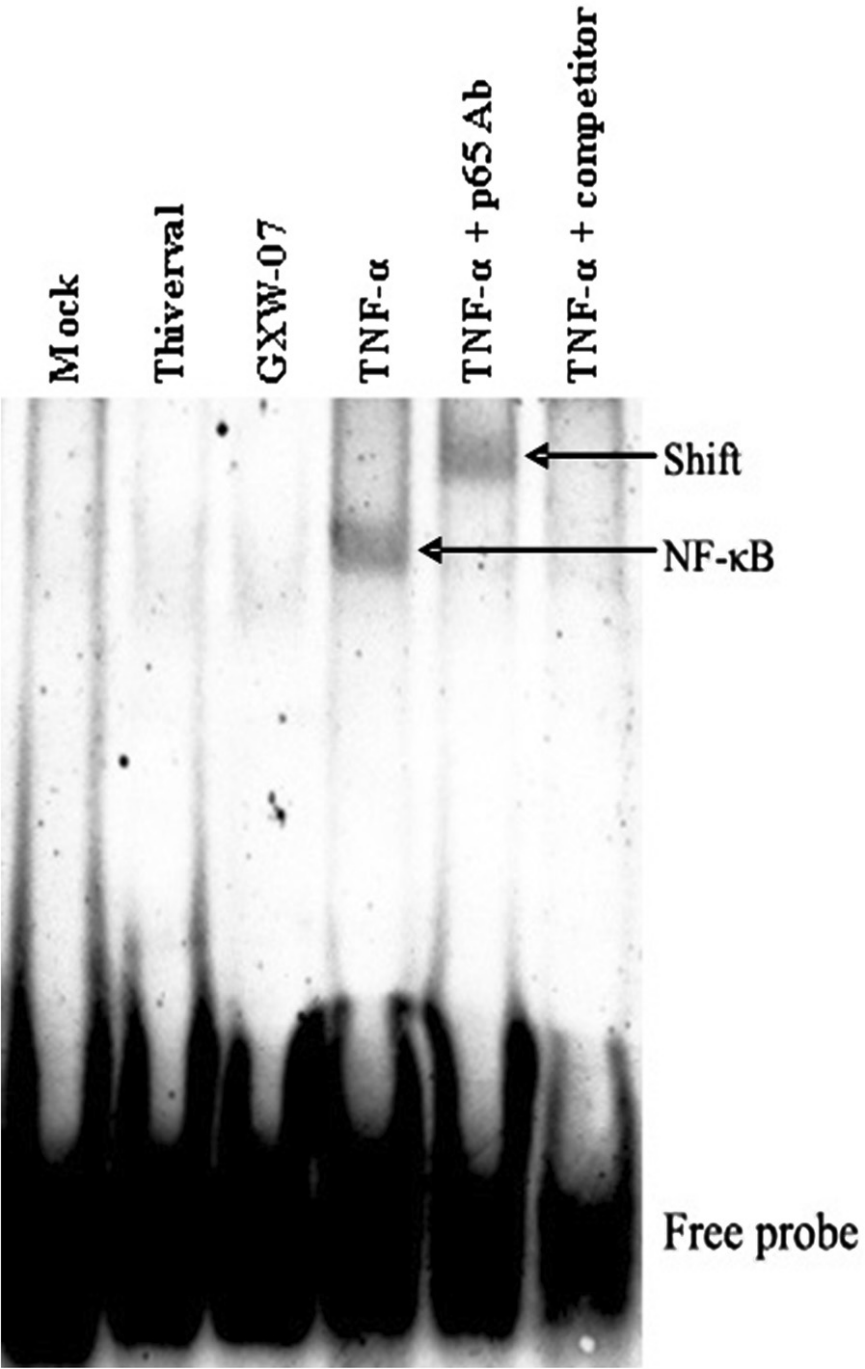
**Analysis of DNA binding activity of NF-κB in CSFV-infected PK-15 cells using electrophoretic mobility shift assays (EMSA).** Cells were mock treated, and TNF-α (10ng/mL) stimulated, or infected with either Thiverval or GXW-07 at an MOI of 1 at 48 h p.i. in PK-15 cells. Nuclear extracts were prepared and subjected to EMSA with a Digoxin labeled NF-κB probe. Anti-p65 rabbit polyclonal antibody was used in supershift assay. A 100-fold molar excess of unlabeled NF-κB probe (unlabeled competitor) was added to analyze the specific bands. The experiment was repeated three times and the figure shows a representative experiment.

**Figure 2:**
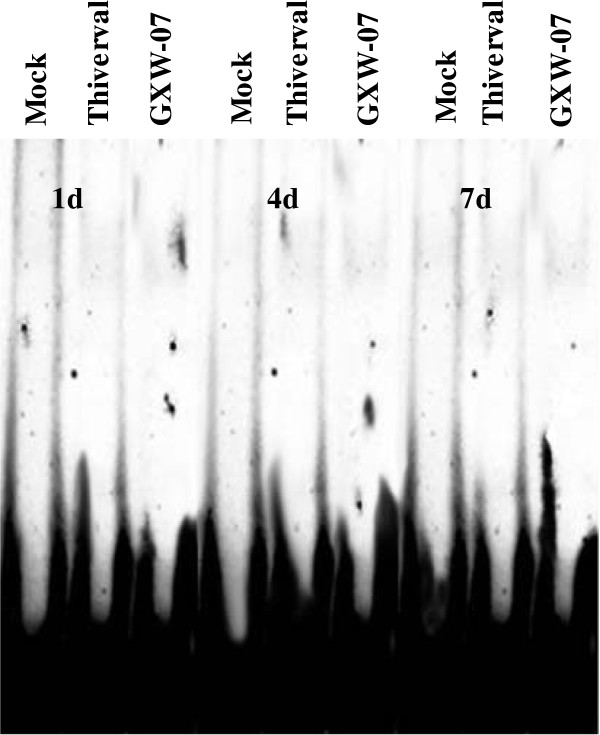
**DNA binding activity of NF-κB in CSFV-infected PBMCs.** Cells treated with Mock, LPS (10ng/mL), Thiverval or GXW-07 at an MOI of 1 were obtained from pigs at 1, 4 and 7d, respectively. Nuclear extracts were prepared and subjected to EMSA with a Digoxin labeled NF-κB probe. Anti-p65 rabbit polyclonal antibody was used in supershift assay. A 100-fold molar excess of unlabeled NF-κB probe (unlabeled competitor) was added to analyze the specific bands. The experiment was repeated three times and the figure shows a representative experiment.

In order to analyze whether CSFV interferes with NF-κB activity, EMSA was performed using the nuclear extracts of PK-15 cells and PBMCs. PK-15 cells cultured *in vitro* were mock treated, TNF-α (10ng/mL) stimulated, and infected with either Thiverval or GXW-07 strain for 48 h. PBMCs collected from the blood of pigs injected with 1 ml physiological saline, Thiverval or GXW-07 strain at 1, 4, 7 d after treatment, respectively. As shown in Figure 1, there was a distinct DNA binding band of NF-κB in TNF-α stimulated cells compared to mock-treated cells that functioned as a negative control. In contrast, neither Thiverval infected cells nor GXW-07 infected cells showed any NF-κB DNA binding activity. A supershift band was observed in TNF-α plus anti-p65 polyclonal antibody treated cells, suggesting the p65 subunit was present in the NF-κB complex. In addition, the shifted band of TNF-α stimulated cells was abolished if cells were treated with 100-fold excess of unlabeled competitor. Results in Figure 2 also revealed that CSFV did not induce DNA binding activity of NF-κB in PBMCs.

### NF-κB p65 translocation in CSF- infected PK-15 cells using immunofluorescent staining

**Figure 3:**
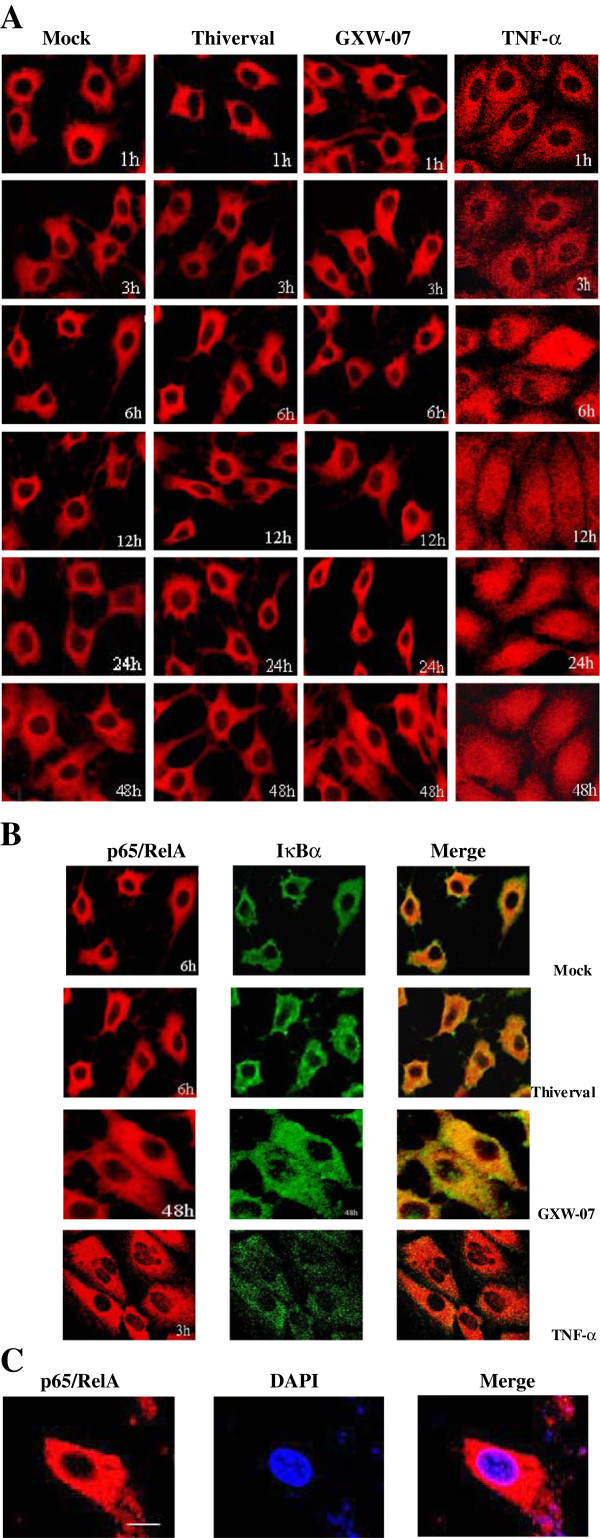
**Effect of CSFV infection on NF-κB translocation in PK-15 cells. A**: Indirect immunofluorescence analysis was used to measure cellular localization of p65/RelA subunit of NF-κB in CSFV-infected PK-15 cells. Cells were mock treated (column 1), and TNF-α (10ng/mL) stimulated (column 4), or infected with Thiverval (column 2) and GXW-07 (column 3) at an MOI of 1. At different periods of time p.i, cells were fixed and the localization of p65/RelA (red) was observed by fluorescence microscope using immunofluorescence staining with anti-NF-κB/p65 and QDs-SA 605-conjugated and biotinylated secondary antibodies. **B**: Co-localization of p65/RelA (red) and IκBα (green) was observed by confocal microscopy. **C**: Nuclei were stained with DAPI. Bar, 10μm. The experiment was repeated three times and the figure shows a representative experiment.

We investigated the NF-κB activation in CSFV-infected PK-15 cells to examine the cellular localization of p65/RelA using a polyclonal anti-p65 antibody. Results are shown in Figure 3. As expected, p65/RelA was predominantly localized in the cytoplasm of mock-treated cells (Figure 3A, column 1). Furthermore, in CSFV-infected cells (Figure 3A, column 2, 3), p65/RelA also remained predominantly in the cytoplasm, although a few cells occasionally showed nuclear localization, indicating that NF-κB remained inactive. Meanwhile, cellular co-localization of IκBα and p65/RelA was detected using confocal microscopy. In contrast, the translocation of p65/RelA in the nucleus was detected in TNF-α stimulated cells at 6h post-stimulation (h.p.s) and p65/RelA further emerged from 12 to 48 h.p.s (Figure 3A, column 4). As shown in Figure 3B, the IκBα protein was also observed predominantly in cytoplasm. The co-localization of IκBα protein with the p65 protein in the cytoplasm was indicated by yellow color in merged images. Additionally, the observation of nuclear staining with DAPI in CSFV-infected cells showed that p65/RelA was specifically located in cytoplasm (Figure 3C). The results above indicated that infection with CSFV did not generate an effective signal for NF-κB nuclear translocation.

### p65/RelA and IκBα expression measured by western blotting

**Figure 4:**
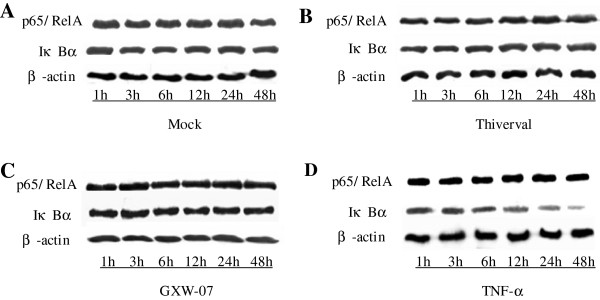
**p65/RelA and IκBα expression in CSFV-infected PK-15 cells within 48 h. A**: PK-15 cells were mock treated. **B**: PK-15 cells were infected with CSFV Thiverval strain at an MOI of 1. **C**: PK-15 cells were infected with CSFV GXW-07 strain at an MOI of 1. **D**: PK-15 cells were TNF-α (10ng/mL) stimulated. In four treatments, extracts of *circa* 20 μg total cells were prepared and subjected to Western Blot analysis with antibodies specific to p65/RelA and IκBα within 48 h p.i. Anti-β-actin served as an internal control. The experiment was repeated three times and the figure shows a representative experiment.

**Figure 5:**
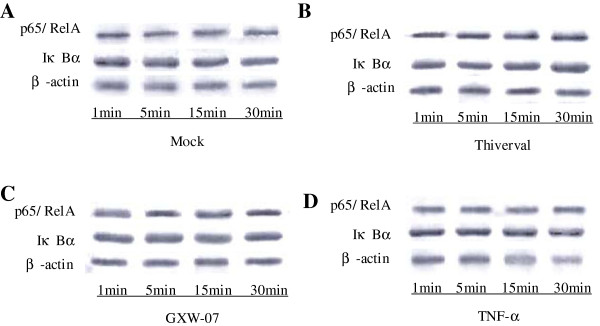
**Western Blot analysis of p65/RelA and IκBα in CSFV-infected PK-15 cells within 1 h. A**: PK-15 cells were mock treated. **B**: PK-15 cells were infected with CSFV Thiverval strain at an MOI of 1. **C**: PK-15 cells were infected with CSFV GXW-07 strain at an MOI of 1. **D**: PK-15 cells were TNF-α (10ng/mL) stimulated. At 1, 5, 15, 30 min p.i, extracts of *circa* 20 μg total cell were prepared and subjected to Western Blot analysis with antibodies specific to p65/RelA and IκBα. Anti-β-actin served as an internal control. The experiment was repeated three times and the figure shows a representative experiment.

**Figure 6:**
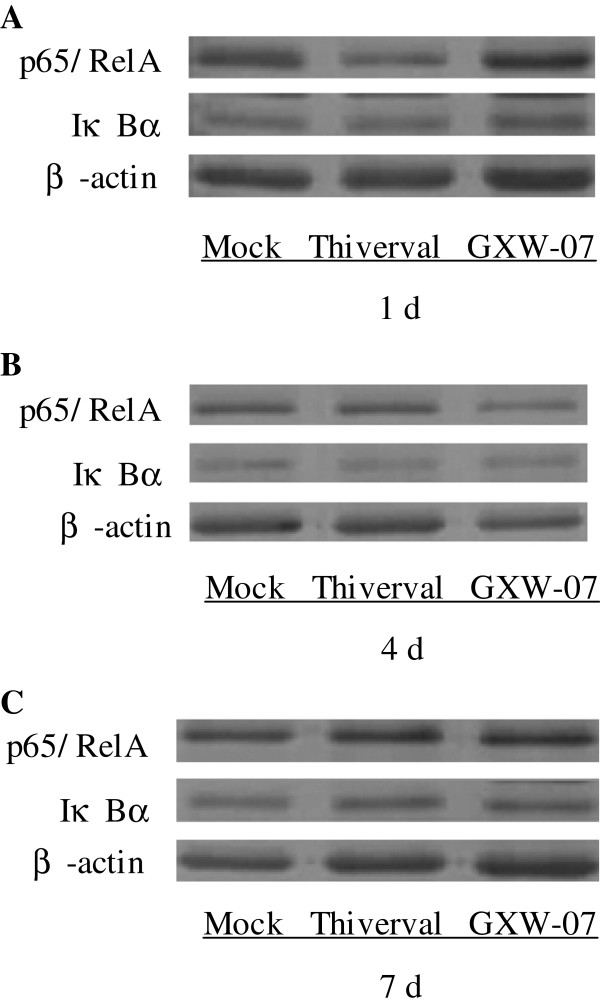
**Effect of CSFV on p65/RelA and IκBα expression in PBMCs. A**: p65/RelA and IκBα expression was measured by Western Blot analysis at 1 d p.i. in mock-treated cells, Thiverval or GXW-07-infected PBMCs. **B**: p65/RelA and IκBα expression was measured by Western Blot analysis at 4 d p.i. in mock-treated cells, Thiverval or GXW-07-infected PBMCs. **C**: p65/RelA and IκBα expression was measured by Western Blotting analysis at 7 d p.i. in mock-treated cells, Thiverval or GXW-07-infected PBMCs. Anti-β-actin served as an internal control. The experiment was repeated three times and the figure shows a representative experiment.

Western Blotting was performed to detect the expression of p65/RelA and IκBα. The results are shown in Figures 4, 5 and 6. Compared to the control (Figure 4A, top panel), no significant change appeared in p65/RelA expression in CSFV-infected PK-15 cells (Figure 4B and C, top panel) at different periods of time after infection, suggesting that CSFV did not alter the expression of p65/RelA. There was also no significant change in the level of p65/RelA in TNF-α-stimulated cells (Figure 4D, top panel). Contrary to p65/RelA, cells treated with TNF-a showed a significant reduction of IκBα expression in a time-dependent manner (Figure 4D, middle panel) compared to that of a basal amount of IκBα in the mock-infected cells (Figure 4A, middle panel), suggesting that degradation of IκBα had occurred. Although TNF-α induced IκBα degradation at 1h p.s, CSFV failed to degrade IκBα at the time points from 1 to 48 h p.i (v 4B and C, middle panel). The activation of NF-κB is a rapid event that occurs within minutes after stimulation. In order to further confirm the observation in Figure 4, the expression of p65/RelA and IκBα in CSFV-infected cells within 1 hour was also detected. As shown in Figure 5A-D, an accordant appearance was obtained. In addition, the level of β-actin did not alter in all conditions (Figure 5 and 6, low panel), indicating no significant difference among the samples. The results in PK-15 cells were also obtained in PBMCs isolated from the blood of pigs at 1, 4 or 7 d after CSFV injection (Figure 6A-D). Our results indicated that the CSFV infection could not activate NF-κB in PK-15 cells and PBMCs because of the failure of IκBα degradation.

## Discussion

The interactions between virus and host may lead to activation or inhibition of NF-κB pathway, resulting in either antiviral responses or enhanced viral replication and virulence [25]. In this report, we investigated the effects of CSFV infection on NF-κB activity in the host using *in vitro* and *in vivo* cell models. Our findings suggest that CSFV infection is incapable of activating NF-κB pathway, as demonstrated by the failure in inducing DNA binding activity of NF-κB, and in causing the translocation of p65/RelA from cytoplasm to the nucleus or the degradation of inhibitor IκBα.

NF-κB plays a crucial role in the process of viral infection [18]. Many viruses activate NF-κB to use it as a transcriptional factor to express viral genes [26]. For example, NF-κB activation appears to be necessary for fully efficient HSV replication and the transcription of viral genes which have NF-κB response elements in their promoters [12,27,28]. NF-κB, however, is potentially dangerous to virus. The activation of NF-κB during viral infection has been considered to be a protective response of the host to the viral pathogens [11,29]. Thus, viruses must subtly control the timing and duration of NF-κB activation to ensure its usefulness as a transcription factor while restricting its antiviral effects [30]. In contrast, some viruses have evolved strategies to block NF-κB activation in order to evade the innate immune response. Human cytomegalovirus encodes a hIL-10 homolog to inhibit the NF-κB transcriptional activity [15]. African swine fever virus encodes an IκBα homolog to inhibit the NF-κB pathway by acting as a dominant-negative IκBα protein [17]; Foot-and-mouth disease virus encodes L protein to disrupt the integrity of NF-κB [16]; Torque teno virus ORF2 protein can suppress NF-κB activity in a dose-dependent manner [18] and Varicella-zoster virus can inhibit the NF-κB pathway by sequestering p50 and p65 in the cytoplasm of infected cells [14]. However, these authors provide no evidence in *in vivo* model. Our results both *in vivo* and *in vitro* show that CSFV has no ability to activate NF-κB, revealing the important ability of CSFV to evade the host immune response and to maintain the life cycle of the virus so as to establish persistent infection, which are consistent to the reports of other researchers [5,6]. Additionally, we find that wild-type or attenuated CSFV strain used in this study shows no difference in activating the NF-κB signaling pathway in PK-15 cells and PBMCs, indicating CSFV with different virulence exerts its influence on NF-κB activity in the same way after it infects the cells.

Viral infection blocks NF-κB activity through various mechanisms [11]. Our study *in vitro* and *in vivo* further demonstrates that the potential mechanism of inability of NF-κB directly depends on the stabilization of IκBα levels. IκBα binds to nuclear localization signal motifs in p50 and p65 to inhibit their nuclear translocation, and the stabilization of IκBα in CSFV-infected cells accounts for the cytoplasmic sequestration of p65/RelA [31]. In most cases, inhibitor IκB irreversibly binds to viral proteins or interferes with IκB degradation, resulting in the inhibition of p65/RelA nuclear translocation [11]. Interestingly, a recent research has described that the N^pro^ product of CSFV had the ability to interact with IκBα and exhibited transient accumulation of pIκBα [6]. Similarly, Gil et al. (2006) reported that the infection of non-cytopathic BVDV (ncp BVDV) together with CSFV could block NF-κB activation. N^pro^ of BVDV was also involved in NF-κB inhibition, but the N^pro^ activity was not essential for NF-κB inhibition [32]. The detailed molecular mechanisms and viral components underlying the suppression of NF-κB activation remain to be elucidated. The ability of CSFV to avoid activating NF-κB to optimize its replication or to control host cell proliferation and survival, the understanding of the molecular mechanisms utilized by the pathogen to interfere with the NF-kB pathway, may enable us to exploit NF-κB as a new weapon against viral diseases. Similar result was described by Santoro et al. (2003) [11].

## Conclusions

In conclusion, we identify that CSFV infection cannot activate the NF-κB signaling pathway *in vitro* and *in vivo*. This mechanism might be utilized by the virus to escape immune rejection and allow the spread of infection. Therefore, understanding the role of NF-κB following CSFV infection would contribute important information about the molecular pathogenesis of CSFV infection.

## Methods

### CSFV preparation

CSFV GXW-07 strain (wild-type strain) and attenuated Thiverval strain were used in this study. The virulent wild-type CSFV GXW-07 strain was originally isolated from a lymphoid tissue sample of a pig with naturally occurring CSF and propagated in PK-15 cells, and its sequence had been analyzed and submitted to GeneBank (No. HQ380231.1). Attenuated Thiverval strain was obtained from the virulent CSFV Alfort strain which was sub-cultured at 29-30°C in PK-15 cells for 170 generations. Virus titres were determined and calculated as described previously [33].

### Cell culture

PK-15 cells used for viral infection were preserved and propagated in our laboratory (Laboratory of Microbiology and Immunology, College of Veterinary, South China Agricultural University). The cells were maintained in Dulbecco’s modified Eagle’s medium (DMEM) supplemented with 10% (v/v) fetal calf serum (FCS), 100 U of penicillin/mL and 100 U of streptomycin/ml at 37°C in a humidified 5% CO_2_ incubator and seeded one day prior to viral infection. Then PK-15 cell monolayer was mock treated, or infected with either CSFV Thiverval strain or GXW-07 strain at multiplicity of infection (MOI) of 1. As a positive control, PK-15 cells were treated with 10 ng/ml TNF-α (R&D Systems).

### PBMC isolation after CSFV infection

CSFV RNA and CSFV antibody were measured respectively using RT-PCR and antibody assay kit in 50-day-old Landrace pigs to choose negative pigs. Then three negative pigs in each group were intramuscularly injected with l ml CSFV Thiverval strain or GXW-07 strain (10^5^TCID50/ ml), and pigs intramuscularly injected with l ml physiological saline were used as a negative control. At 1, 4 and 7d p.i., 10 ml blood from each pig was collected in sodium heparin-CPT tubes and diluted with an equal volume of phosphate buffered saline (PBS). Then 5 ml diluent was carefully loaded into tubes containing 5 ml lydroxypropylmethyl cellulose and centrifuged at 1500 rpm for 20 minutes at room temperature. The buffy coat layer was transferred to a 15 ml RNAse-free tube, diluted with an equal volume of PBS, and centrifuged at 1500 rpm for 20 minutes at room temperature. The supernatant was discarded and PBMCs were diluted with 9 ml red cell lysate, and centrifuged at 1000 rpm for 10 minutes at room temperature after ice bath for 2-3 min. The supernatant was discarded and PBMCs were diluted with PBS for three times. PBMCs were retained and used to detect NF-κB activity.

The *in vivo* experiment was carried out according to the recommendations in the Guide for the Care and Use of Laboratory Animals of the National Institutes of Health. The protocol was approved by Medical Experimental Animal Center of Guangdong Province (Permit Number: 12-179). All surgery was performed under sodium pentobarbital anesthesia, and all efforts were made to minimize suffering.

### Electrophoretic mobility shift assays (EMSA)

PK-15 cells mock treated, TNF-α (10ng/mL) stimulated, or infected with either Thiverval strain or GXW-07 strain were harvested for 48 h, and nuclear protein extracts were prepared with the nuclear extraction kit (Pierce, USA). The protein concentration of the cell extract was measured by the Bradford assay (Pierce, USA). Activation of p65/IκBα was determined by EMSA (Roche, USA) according to the manufacturer's manual. A DIG-labeled double-stranded oligonucleotide containing a κB binding site (GGGACTTTCC) was used as a probe in the assay. Anti-p65 rabbit polyclonal antibodies (Neomarker, USA) were used in supershift assays. For competition assays, a 100-fold molar excess of unlabeled oligonucleotide was added. DNA binding activity of NF-κB in PBMCs obtained from infected pigs was also determined in the same way mentioned above.

### Immunofluorescent staining

Subcellular localization of p65/IκBα was determined by immunofluorescencent staining. At different time points, cultured PK-15 cells in four treatments were washed with PBS, fixed in 80% acetone for 10 min, and permeabilized for internal staining with PBS containing 0.1% Triton X-100 (Sigma, USA) for 30 min. The cells were then blocked in TBS containing 3% BSA (Sigma, USA) for 30 min and incubated with rabbit polyclonal anti-p65 (diluted 1:100; Neomarker, USA) or anti-IκBα (diluted 1:500; Santa Cruz, USA) antibodies diluted in 1% BSA-TBS at 37°C for 2-3 h. After washed three times with TBS, the cells were further incubated with either QDs-SA 605 (excitation wavelength of ultraviolet light and fluorescent emission 605 nm, red fluorescent) or QDs-SA 545 (excitation wavelength of ultraviolet light and fluorescent emission 545nm, green fluorescent) (Molecular Probes, Jiayuan Quantum Dots, Wuhai, China)-conjugated and biotinylated secondary antibodies at a 1:200 dilution in TBS/1% BSA at 37°C for 30 min. Then cells were stained with DAPI for 10 min and washed twice in TBST and TBS respectively. The coverslips were mounted onto microscope slides, and cells were visualized using a Leica SP2 confocal scanning laser microscope (Leica) with the appropriate barrier filters.

### Western blot analysis

Cells were lysed in Cell Lysis Buffer (Cell Signaling, USA) supplemented with 1 mM phenylmethylsulfonyl fluoride (PMSF) for 30min on ice and clarified by centrifugation for total protein extraction. Protein concentration was quantified as described in EMSA. *Circa* 20 μg of proteins was separated by 10% SDS-polyacrylamide gel electrophoresis (SDS-PAGE) and transferred to nitrocellulose (NC) membranes. The membranes were blocked in TBST containing 5% powdered milk for 3 h and incubated overnight with primary antibodies at 4°C. Blots were washed and probed with secondary antibody at 37°C for 1 h. The membranes were then developed with enhanced chemiluminescence substrate (Amersham) and exposed to X-ray. As a control, the gels were stripped and re-probed with antibodies against β-actin. Polyclonal anti-NF-κB/p65 (diluted 1:200; Neomarker, USA), polyclonal anti-IκBα (diluted 1:1000; Santa Cruz, USA), polyclonal anti-β-actin (diluted 1:500; Boisynthesia, China) and HRP-conjugated anti-rabbit secondary antibody (diluted 1:1000; Santa Cruz, USA) were used in this study.

## Abbreviations

BDV: Border disease virus; BVDV: Bovine viral diarrhoea virus; CSFV: Classical swine fever virus; DMEM: Dulbecco’s modified Eagle’s medium; ECL: Enhanced chemiluminescence; EMSA: Electrophoretic mobility shift assays; FCS: Fetal calf serum; IFN: Type I interferon; Mo-DCs: Monocyte-derived dendritic cells; NC: Nitrocellulose; NF-κB: Nuclear factor-kappa B; PBMCs: Peripheral blood mononuclear cells; PBS: Phosphate buffered saline; Pi: Post-infection; PK-15: Porcine kidney 15; PMSF: Phenylmethylsulfonyl fluoride; SDS-PAGE: SDS-polyacrylamide gel electrophoresis; TNF-α: Tumor necrosis factor-alpha.

## Competing interests

The authors declare that they have no competing interests.

## Authors’ contributions

LJC and XYD contributed equally to this work; they designed this study, carried out EMSA analysis and drafted the manuscript; MQZ undertook CSFV infection and participated in the interpretation of data; JJP and WJL carried out Western Blot analysis; JYW and YWL bred the animals and isolated PBMCs; CMJ and HYS cultured PK-15cells and performed immunofluorescent staining analysis; as the corresponding author of this study, JDC participated in study design and revised the manuscript. All authors read and approved this version to be published.

## Authors’ information

LJ Chen and HY Shen have a PhD in Preventive Veterinary Medicine and have been working on cell signaling pathway induced by viral infection; XY Dong and WJ Liu are involved in evaluation of immune responses after vaccination in virus-infected animals; MQ Zhao and YW Luo study transmission of CSFV and vaccine responses after viral infection; JJ Pei and JY Wang carry out the investigation in cell apoptosis and autophagy after viral and bacterial infection; CM Ju has a PhD in Preventive Veterinary Medicine and investigates pig diseases; JD Chen holds a PhD in Preventive Veterinary Medicine and works on immunology and microbiology. He also makes investigation into cell signaling pathway after viral infection.

## References

[B1] Becher P Thiel HJ Tidona CA, Darai G Genus Pestivirus (Flaviviridae) The springer index of viruses 2002 Heidelberg, Germany: Springer 327 331

[B2] Summerfield A Knoetig SM McCullough KC Lymphocyte apoptosis during classical swine fever: implication of activation-induced cell death J Virol 1998 72 1853 1861 9499036 10.1128/jvi.72.3.1853-1861.1998 PMC109475

[B3] Jamin A Gorin S Cariolet R Potier MFL Kuntz-Simon G Classical swine fever virus induces activation of plasmacytoid and conventional dendritic cells in tonsil, blood, and spleen of infected pigs Vet Res 2008 39 7 10.1051/vetres:2007045 18073094

[B4] Sato M Mikami O Kobayashi M Nakajima Y Apoptosis in the lymphatic organs of piglets inoculated with classical swine fever virus Vet Microbiol 2000 75 1 9 10.1016/S0378-1135(00)00198-X 10865147

[B5] Charleston B Fray MD Baigent S Carr BV Morrison WI Establishment of persistent infection with non-cytopathic bovine viral diarrhoea virus in cattle is associated with a failure to induce type I interferon J Gen Virol 2001 82 1893 1897 11457995 10.1099/0022-1317-82-8-1893

[B6] Doceul V Charleston B Crooke H Reid E Powell PP Seago J The Npro product of classical swine fever virus interacts with IκBα, the NF-κB inhibitor J Gen Virol 2008 89 1881 1889 10.1099/vir.0.83643-0 18632959

[B7] Bensaude E Turner JLE Wakeley PR Sweetman DA Pardieu C Drew TW Wileman T Powel PP Classical swine fever virus induces proinflammatory cytokines and tissue factor expression and inhibits apoptosis and interferon synthesis during the establishment of long-term infection of porcine vascular endothelial cells J Gen Virol 2004 85 1029 1037 10.1099/vir.0.19637-0 15039545

[B8] Ruggli N Bird BH Liu L Bauhofer O Tratschin JD Hofmann MA Npro of classical swine fever virus is an antagonist of double-stranded RNA-mediated apoptosis and IFN-α/β induction Virology 2005 340 265 276 10.1016/j.virol.2005.06.033 16043207

[B9] Amici C Rossi A Costanzo A Ciafre S Marinari B Balsamo M Levrero M Santoro MG Herpes simplex virus disrupts NF-κB regulation by blocking its recruitment on the IκBα promoter and directing the factor on viral genes J Bio Chem 2006 281 7110 7117 10.1074/jbc.M512366200 16407234

[B10] Pahl HL Activators and target genes of RelA/NF-κB transcription factors Oncogene 1999 18 6853 6866 10.1038/sj.onc.1203239 10602461

[B11] Santoro MG Rossi A Amici C NF-kappa B and virus infection: who controls whom EMBO J 2003 22 2552 2560 10.1093/emboj/cdg267 12773372 PMC156764

[B12] Gregory D Hargett D Holmes D Money E Bachenheimer SL Efficient replication by herpes simplex virus type 1 involves activation of the IκB kinase-IκB-p65 pathway J Virol 2004 78 13582 13590 10.1128/JVI.78.24.13582-13590.2004 15564469 PMC533927

[B13] Indoh T Yokota S Okabayashi T Yokosawa N Fujii N Suppression of NF-κB and AP-1 activation in monocytic cells persistently infected with measles virus Virol 2007 361 294 303 10.1016/j.virol.2006.11.002 17196632

[B14] Jones JO Arvin AM Inhibition of the NF-κB pathway by varicella-zoster virus in vitro and in human epidermal cells in vivo J Virol 2006 80 5113 5124 10.1128/JVI.01956-05 16698992 PMC1472140

[B15] Nachtwey J Spencer JV HCMV IL-10 Suppresses cytokine expression in monocytes through inhibition of nuclear factor-κB Viral Immunol 2008 21 477 482 10.1089/vim.2008.0048 19115937 PMC2831744

[B16] Santos T Segundo FDS Grubman MJ Degradation of nuclear factor kappa B during foot-and-mouth disease virus infection J Virol 2007 81 12803 12815 10.1128/JVI.01467-07 17881445 PMC2169123

[B17] Silk RN Bowick GC Abrams CC Dixon LK African swine fever virus A238L inhibitor of NF-κB and of calcineurin phosphatase is imported actively into the nucleus and exported by a CRM1-mediated pathway J Gen Virol 2007 88 411 419 10.1099/vir.0.82358-0 17251557

[B18] Zheng H Ye L Fang X Li B Wang Y Xiang X Kong L Wang W Zeng Y Ye L Wu Z She Y Zhou X Torque teno virus (SANBAN Isolate) ORF2 protein suppresses NF-κB pathways via interaction with IκB kinases J Virol 2007 81 11917 11924 10.1128/JVI.01101-07 17686849 PMC2168763

[B19] Lin S Wu M Xu Y Xiong W Yi Z Zhang X Zheng HY Inhibition of hepatitis B virus replication by MyD88 is mediated by nuclear factor-kappaB activation Biochim Biophys Acta 2007 1772 1150 1157 10.1016/j.bbadis.2007.08.001 17935950

[B20] Lin YC Hsu EC Ting LP Repression of hepatitis B viral gene expression by transcription factor nuclear factor-kappaB Cell Microbiol 2009 11 645 660 10.1111/j.1462-5822.2008.01280.x 19141126

[B21] Moon DO Kim MO Kang SH Choi YH Kim GY Sulforaphane suppresses TNF-a-mediated activation of NF-κB and induces apoptosis through activation of reactive oxygen species-dependent caspase-3 Cancer Lett 2009 274 132 142 10.1016/j.canlet.2008.09.013 18952368

[B22] Ortis F Pirot P Naamane N Kreins AY Rasschaert J Moore F Théâtre E Verhaeghe C Magnusson NE Chariot A Ørntoft TF Eizirik DL Induction of nuclear factor-κB and its downstream genes by TNF-α and IL-1β has a pro-apoptotic role in pancreatic beta cells Diabetologia 2008 51 1213 1225 10.1007/s00125-008-0999-7 18463842

[B23] Brown K Gerstberger S Carlson L Franzoso G Siebenlist U Control of IκB-alfa proteolysis by site specific signal induced phosphorylation Science 1995 267 1485 1487 10.1126/science.7878466 7878466

[B24] Atkinson PG Coope HJ Rowe M Ley SC Latent membrane protein 1 of Epstein-Barr virus stimulates processing of NF-kappa B2 p100 to p52 J Bio Chem 2003 278 51134 51142 10.1074/jbc.M304771200 14532284

[B25] Lee SM Kleiboeker SB Porcine arterivirus activates the NF-κB pathway through IκB degradation Virology 2005 342 47 59 10.1016/j.virol.2005.07.034 16129468 PMC7111765

[B26] Shisler JL Jin XL The vaccinia virus K1L gene product inhibits host NF-κB activation by preventing IκBα degradation J Virol 2004 78 3553 3560 10.1128/JVI.78.7.3553-3560.2004 15016878 PMC371086

[B27] Patel A Hanson J McLean TI Olgiate J Hilton M Miller WE Bachenheimer SL Herpes simplex type 1 induction of persistent NF-kappa B nuclear translocation increases the efficiency of virus replication Virology 1998 247 212 222 10.1006/viro.1998.9243 9705914

[B28] Taddeo B Zhang W Lakeman F Roizman B Cells lacking NF-κB or in which NF-κB is not activated vary with respect to ability to sustain herpes simplex virus 1 replication and are not susceptible to apoptosis induced by a replication-incompetent mutant virus J Virol 2004 78 11615 11621 10.1128/JVI.78.21.11615-11621.2004 15479802 PMC523294

[B29] Wei L Kuang J Wang J Shi L Yang B Li Y Liu J Porcine circovirus type 2 induces the activation of nuclear factor kappa B by IκBα degradation Virology 2008 378 177 184 10.1016/j.virol.2008.05.013 18561971

[B30] Hiscott J Kwon H Genin P Hostile takeovers: viral appropriation of the NF-κB pathway J Clin Invest 2001 107 143 151 10.1172/JCI11918 11160127 PMC199181

[B31] Richmond A Nf-kappa B, chemokine gene transcription and tumor growth Nat Rev Immunol 2002 2 664 674 10.1038/nri887 12209135 PMC2668257

[B32] Gil LHVG van Olphen AL Mittal SK Donis RO Modulation of PKR activity in cells infected by bovine viral diarrhea virus Virus Res 2006 116 69 77 10.1016/j.virusres.2005.08.011 16194578

[B33] Knoetig SM Summerfield A Spagnuolo-Weaver M McCullough KC Immunopathogenesis of classical swine fever: role of monocytic cells Immunology 1999 97 359 366 10.1046/j.1365-2567.1999.00775.x 10447754 PMC2326829

